# Closing gaps between open software and public data in a hackathon setting: User-centered software prototyping

**DOI:** 10.12688/f1000research.8382.2

**Published:** 2016-05-09

**Authors:** Ben Busby, Matthew Lesko, August 2015 and January 2016 Hackathon participants, Lisa Federer

**Affiliations:** 1 National Center for Biotechnology Information (NCBI), National Library of Medicine, National Institutes of Health, Bethesda, MD, USA; 2 NIH Library, Division of Library Services, Office of Research Services, National Institutes of Health, Bethesda, MD, USA

**Keywords:** Pharmacogenomics, Bioconductor, Next Generation Sequencing, Genomics, Open-Source, Genome Annotation, Education, Software

## Abstract

In genomics, bioinformatics and other areas of data science, gaps exist between extant public datasets and the open-source software tools built by the community to analyze similar data types.  The purpose of biological data science hackathons is to assemble groups of genomics or bioinformatics professionals and software developers to rapidly prototype software to address these gaps.  The only two rules for the NCBI-assisted hackathons run so far are that 1) data either must be housed in public data repositories or be deposited to such repositories shortly after the hackathon’s conclusion, and 2) all software comprising the final pipeline must be open-source or open-use.  Proposed topics, as well as suggested tools and approaches, are distributed to participants at the beginning of each hackathon and refined during the event.  Software, scripts, and pipelines are developed and published on GitHub, a web service providing publicly available, free-usage tiers for collaborative software development. The code resulting from each hackathon is published at https://github.com/NCBI-Hackathons/ with separate directories or repositories for each team.

## Introduction

The expansion of next-generation sequencing has led to a coming-of-age of specialists seeking to advance the field. For example, researchers collaboratively collect expansive data to define the spectrum of human genetic variation ^1^. Coders develop fast and accurate alignment algorithms to handle the profusion of short reads typifying next-generation sequencing ^2^. Biologists employ novel sequencing techniques to reconstruct mutations arising during cancer progression ^3^.

Ideally, genomics pipelines simultaneously leverage the talents of all of these specialists, as well as combining the capacity of big data, the power of computation and statistical analysis, and the control of experimental design. However, members of one specialty may lack the expertise to fully appreciate the tools - computational or experimental - developed by another specialty, leading to gaps between data, code, and users. Users may also lack the resources to gather annotations scattered across datasets. Collaboration among specialists across the spectrum of data collectors, generators, coders, and users provides an opportunity for feedback and moves the field toward closing gaps in accessibility of tools. Unfortunately, the daily routine and structure of research communities do not always facilitate these exchanges. We propose the hackathon format as a solution to breaking down these barriers, providing a unique opportunity to gather “hackers” from diverse backgrounds to work together on focused challenges and create novel solutions to real-world problems.

The purpose of this article, and, more broadly, this channel, is to provide a space to announce software prototypes developed in biological data science hackathons, as well as to inform and assist in the creation of such hackathons. Anyone who has developed and run such a hackathon is invited to contribute to this evolving editorial.

### Events to date, community context and goals

As of this publication, we have hosted three community hackathons, assisted by the National Center for Biotechnology Information (NCBI). The first three events were held at the National Institutes of Health (NIH) in January 2015, August 2015, and January 2016, and attended by over 110 participants from 5 countries. The results of the groups participating in the January 2015 hackathon were collected in a single paper and have been posted to bioRxiv ^4^. For subsequent hackathons, groups have reported their findings individually; discussions of their results are available in this channel, as well as in other forthcoming publications.

## Designing “anabolic” hackathons

There are several kinds of hackathons. The term is frequently associated with competitive hackathons, in which teams compete against each other for a “best” solution, typically with a prize awarded to the winning teams. Groups running such hackathons are welcome to post their designs and results to this space. However, the hackathons we are running in 2015/2016 are primarily what we have termed “anabolic” hackathons. In an anabolic hackathon, each team builds -- or builds onto -- a different project, instead of competing against each other on the same project. Rather than viewing fellow hackathon participants as competitors, participants in these anabolic hackathons frequently work together with other teams to solve problems and propose solutions.

### Hosting and preparing for anabolic hackathons

Hackathons are often most successful when they are able to gather a group of individuals representing a wide diversity of opinions and skills. Therefore, we have selected sites in order to provide maximal regional diversity and to accommodate as many different participants with varied backgrounds as possible. In addition to the hackathons at NIH, NCBI also provides assistance for regional hackathons and seeks applications from local organizers interested in hosting such events. Minimally, the site must be able to provide adequate computational resources (either locally or through a cloud provider). Sites are selected based on their diversity plan and whether the site is located in close regional proximity to other recent hackathon sites. Local hosts interested in applying to host a hackathon with NCBI assistance should complete the online form at https://goo.gl/ZB0UyV.

In advance of the hackathon, the organizers select scientific problems and potential approaches to solving them, based on common bioinformatics and genomics use cases that involve public datasets. Potential team leads, who are subject matter experts in fields of significance to a selected scientific problem, are approached with an approximate plan for how their team might approach the problem within the scope of a three-day hackathon. Project team leads are often NCBI and NIH personnel, but may also be outside collaborators invited based on their known expertise in the field.

The organizer and team leaders typically discuss several (4–8) iterations of the proposed plan in advance of the hackathon. During these iterations, an approximate title of the project is generated for the event announcement. Announcements ( example here) are then released to solicit applications; for hackathons thus far we have received three to seven times more applicants than we could accommodate in the hackathon. After accepting applications for approximately two weeks, the applications are distributed to team leads, who select 4–6 people with their teams, plus an alternate. After the membership of all teams is finalized, the organizer completes and distributes logistical schedules and scientific schedules. Before the hackathon, emails are distributed to briefly introduce team members to each other. Participants are notified of their team’s general scientific problem, but not the specific projects they will be addressing, in order to prevent solo hacking in advance of the event.

### Logistics

In the process of creating a set of deliverables over the course of the hackathon, we have observed that teams become proficient with collaborative development tools, such as Git ^5^ and GitHub for version control and software development, Gitter for team chats and conversations ^6^, Amazon Simple Storage Service (AWS S3) for file sharing ^7^, and individual Google Groups for general team communication ^8^. Markdown documents, supported by GitHub, are used by most teams for documentation, though groups have also used the GitHub wiki system ^9^. Cloud compute is often used for these events. An additional description of the specifics of these solutions is contained in the Supplementary material.

The organizers do little to direct team management strategies and we have noticed that these have varied across groups as well as over time within groups. Despite the ad hoc nature of group formation, only two participants have changed teams, and only one has ever left a hackathon. Working lunches, during which each group presents progress in a town-hall style fashion, occur each day. At other times, break out rooms are provided so that teams can work individually or in smaller sub-groups. Though the hackathons were originally scheduled to run over the course of the business day, we have considered providing spaces for hacking in the evenings during future events, due to participants’ enthusiasm and desire to continue working on their problems once the official day is over.

We also emphasize the importance of each member of every group contributing to documentation of the hackathon. Documentation includes software report manuscripts submitted to journals, including this one, as well as markdown or wiki documentation on GitHub. All the members of each group contribute to documentation, which is compiled by an individual from each group who has been appointed as the group’s writer, with additional contribution from and editing by a librarian participating in the event. In addition to the writer role, future hackathon groups will also have an individual appointed to the role of advertiser, responsible for coming up with a plan to promote the resulting pipeline and encouraging its use in the scientific community.

## Outcomes

### Software products

**Figure 1.**
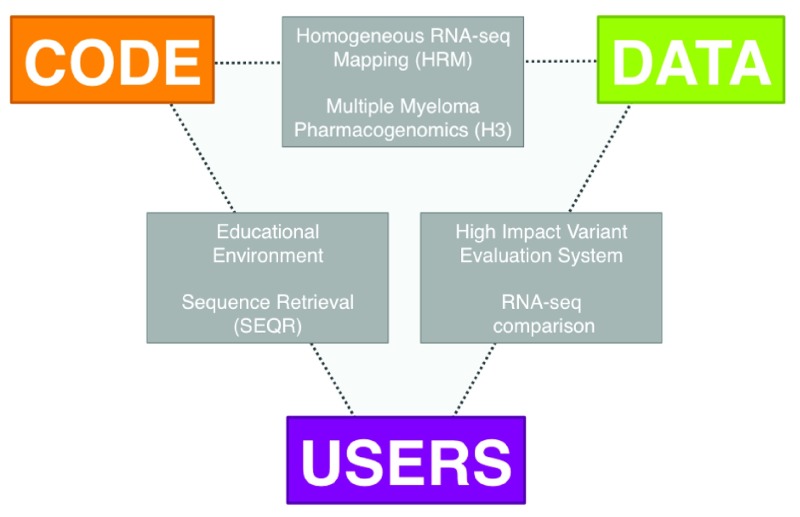
Outline of the pipeline in Homogeneous RNA-seq Mapping (HRM) team. The leftmost column shows procedures and the next columns are tools used in each step and files created by each tool, respectively. HISAT directly accesses SRA data of interest for users and provides aligned reads in a SAM file. Picard classified reads sorted by SAMtools into functional categories using the RefFlat file. After the quality check by qc.pl, HTSeq calculates raw read counts at each region.

The main objective of our hackathons is to shorten the distance between users, genomic data, and the computational tools necessary to analyze that data (see Figure 1 for an example diagram, generated during the NCBI August 2015 hackathon). We emphasize deliverables that facilitate this interaction; most teams provide example pipelines that implement their approaches. Synopses for the resulting tools, along with suggestions of how these tools may be used, are published individually.

### Additional benefits of participation

Our hackathons conclude with 15 minute presentations from each of the groups. During these presentations, most groups have expressed interest in continuing to work on their projects until completion, and many also expressed a desire to add further refinements and features as well. We believe that this commitment to completing their projects, even after the end of the hackathon, reflects a generally positive experience among participants from the hackathon.

Specifically, many hackathon participants expressed that they benefited greatly from interactions with new individuals from different fields of expertise and with varying types of knowledge. Working in teams accelerated conversations between groups that often don’t talk together about fixing the gaps that slow down this type of research. Coders had to immediately answer to data generators: “what’s the input?” and “what’s the output?” Data generators had to immediately answer to coders: “who is the audience?” Making tools that were easy to implement and that enabled easy data analysis was a common goal for five of the groups. Additionally, teaching resources and comparative tools for those new to the field have been generated in several events. The type of cross-field collaboration that occurs through hackathons facilitates the creation of these kinds of resources.

Within their groups, each member was allowed to choose which roles to take on and which parts of the problem to work on. As a result, participants felt a sense of ownership over their work and responsibility for pursuing the task without ego. People were interested in learning new strategies and seeing things from a new perspective. Accomplishing a defined task with their team in a short period of time gave the members an almost immediate sense of satisfaction, something very elusive in their day to day jobs.

## Future directions

The next challenge for NCBI hackathons is to engage more community involvement with the software products arising out of the hackathons, both in updating and improving the products as well as creating new modules that build upon the initial work. As with any open software or data source, increased use and awareness of publicly available resources could help create a more unified bioinformatics software community. To this end, the organizers and participants are continuing to work to publicize their software products, both through scholarly literature like the articles available in this channel, and elsewhere.

Based on the success of these initial hackathons, the organizers plan on facilitating similar, regional hackathons at different universities and medical centers across the United States. Given the high level of interest in participating in hackathons, as evidenced by the large number of applicants for each of the previous hackathons, we hope that future hackathons to be similarly popular and successful. In addition, we encourage other members of the community to host their own hackathons and have shared resources for anyone to create a similar event on our GitHub site ^10^.

## Data and software availability

The code for NCBI-assisted hackathon projects is available at https://github.com/NCBI-Hackathons/.

## Supplementary material

Information about computing, storage, and other technical logistics necessary for hackathons.
