# Supplementary material for: Closing gaps between open software and public data in a hackathon setting: User-centered software prototyping
Source: F1000Res. 2016 May 9;5:672. Originally published 2016 Apr 13. [Version 2] doi: 10.12688/f1000research.8382.2 (PMC4837979; doi:10.12688/f1000research.8382.2)
Supplement: Supplementary file 1 [file f1000research-5-9419-s0000.tgz › f0871e79-8074-4058-b398-7309af92db1e.docx]

Supplement One: Description of the Hackathon Compute Environment

A public cloud provider was selected for the hackathon in order to quickly create resources before and during the hackathon and immediately release them after use. Using a public cloud provider also reduced the need for high performance networking to the participants’ individual machines. The cloud infrastructure used was simple, consisting of virtual machines, networking, block storage, and object storage. None of the cloud services were specific to any public cloud provider. Amazon Web Service (AWS) was chosen because of previous operational experience.

# Operating system and software

The base operating system was configured in AWS using Ubuntu 14.04 “Trusty Tahr,” 64-bit Linux as the operating system. “Instance type” (server size) was chosen ahead of time. The type *m4.4xlarge* with 16 virtual CPUs and 30GiB of RAM was considered adequate for most teams. A few teams required larger and/or more numerous instances, which were started on demand as needed. No clustering or resource management (StarCluster, Elasticcluster, Slurm/GCE/Torque) was pre-installed, although some teams were comfortable “hacking together” a cluster using SSH over multiple nodes.

A substantial number of software packages were downloaded, compiled, and installed into: /opt/[software-package-name]. “Profile scripts” were created for each software package, kept in /etc/profile.d/[software-package-name].sh. These scripts added the software package to the user PATH variable so that users could immediately run the packages after logging into the instance.

# Storage

Storage was configured as one terabyte (TB) of Elastic Block Storage (EBS) using general purpose SSDs (gp2) as the singular, root partition. This size was adequate for most teams; only two teams requested additional storage. All of the EBS size was added as the root (/) partition, so that /opt, /data, and /home could also be used without thinking about the data location. The trade-off was reduced flexibility for increased simplicity; no teams had to work with the AWS technical lead to move data to or from a “correct” place. Additionally, an S3 bucket was created solely for the hackathon. An AWS Identity and Access Management (IAM) user was created with API keys and an IAM policy that provided read/write access *only* to that S3 bucket. *All* instances had “s3cmd” installed and configured to use the IAM user API keys. Similar to the ubuntu user decision, below, the expediency of a single location and credentials was perceived as more efficient for a hackathon environment.

Several larger datasets (multiple myeloma and reference genome and annotation) were downloaded ahead of time into /data and saved into the Amazon Machine Image (AMI).

# Security, authentication, and authorization

All instances were started within the same Amazon virtual private cloud (VPC) and placed into the same placement group to provide low-latency network access between instances.  Access was restricted to the private IP addresses within the VPC. An S3 VPC endpoint was created in the VPC to decrease latency and enhance security by only permitting access from the VPC IP addresses. All instances were started with the same security groups - port 22/tcp (SSH) only, open to the world. This decision was made so that hackathon participants could work from different network environments, both on-campus or from home. No other ports were permitted. Users were instructed on how to use “SSH port-forwarding” if they needed to use a remote web server or Solr interactively.

For efficiency it was decided to permit all hackathon users to login as the same user, ubuntu. There are advantages and disadvantages to this approach. An advantage is that team collaboration (sharing data, code) is trivial. A disadvantage is the lack of accountability and potential to disturb teammate’s work. To avoid that we suggested, but did not enforce, that all users create their own private working directory within $HOME. In the experience of this hackathon, the advantages greatly surpassed the disadvantages.

All hackathon participants were asked to register an account with Github (<http://www.github.com>), a free collaborative software development environment. All hackathon participants were asked to upload one or more SSH keys to their Github account, both for Github collaboration and to permit logins to each team’s Amazon Web Service server (instance).

Adding hackathon members to an instance was extremely convenient thanks to a program installed in Ubuntu 14.04, “Trusty Tahr”: ssh-import-id (<http://packages.ubuntu.com/trusty/ssh-import-id>). It takes as a parameter a collaboration website (BitBucket or Github) and a username, and then adds the same public SSH key as a permitted login user. Contrasted with the previous hackathon, this solved a huge administrative and time burden - almost every user came to the hackathon able to login to their servers and begin work. (There were a few exceptions, which were manually solved after the start, but at 3-5 minutes per user, this would have been a burden at 40+ participants, particularly as SSH & SSH keys have their own lingo and learning curve).
